# Central trigeminocardiac reflex in pediatric neurosurgery: a case report and review of the literature

**DOI:** 10.1186/1752-1947-6-372

**Published:** 2012-10-30

**Authors:** Toma Spiriev, Christo Tzekov, Lili Laleva, Christina Kostadinova, Slavomir Kondoff, Nora Sandu, Bernhard Schaller

**Affiliations:** 1 Department of Neurosurgery, Tokuda Hospital Sofia, 51b Nikola Vaptsarov Boulevard, Sofia, 1407, Bulgaria; 2 Department of Anesthesiology, Tokuda Hospital Sofia, Sofia, Bulgaria; 3 Department of Neurosurgery, University of Paris, Paris, France; 4 Department of Neurosurgery, Tokuda Hospital Sofia, Sofia, Bulgaria

## Abstract

**Introduction:**

Trigeminocardiac reflex is a well-known phenomenon in neurosurgery, craniofacial surgery, ophthalmology and interventional neuroradiology. Even though the trigeminocardiac reflex has become an important factor in skull base surgery and neurosurgery, the central form of trigeminocardiac reflex has only been described in adult subpopulations until now.

**Case presentation:**

We present a clear form of repetitive trigeminocardiac reflex expressed during revision surgery of a giant (110×61mm) right temporoparietal meningioma in an 18-month-old male Caucasian patient. After cessation of the surgical stimulus, his heart rate and mean arterial blood pressure returned to normal physiological levels. The further follow-up was uneventful.

**Conclusion:**

Our case demonstrates that the central trigeminocardiac reflex also exists in pediatric patients, especially if manipulating trigeminal innervated structures or around the nerve itself. Whether the incidence and the behavior of the trigeminocardiac reflex is similar in pediatric neurosurgery compared with adult patients has to be shown in further studies.

## Introduction

The trigeminocardiac reflex (TCR) is a well-known brainstem reflex leading to sudden onset of parasympathetic activity, sympathetic hypotension, apnea or gastric hypermotility during central or peripheral stimulation of any of the sensory branches of the trigeminal nerve [1-7]. The peripheral form of TCR is well described in craniofacial surgery as oculocardiac reflex (OCR), and can been seen during ophthalmologic interventions that are associated with stimulation of the ophthalmic division of the trigeminal nerve [7-9]. The clinical importance of OCR, a physiologic variant of TCR, is well described in all age groups and - without routine use of anticholinergic medication - the reported incidence may be as high as 90% [7-9]. The first description of the central form of the TCR was performed by the senior author (BS) during neurosurgical skull base surgery in 1999 [1]. Thereafter, this reflex has been reported in other intracranial neurosurgical procedures, including supratentorial surgeries, surgery around the falx cerebri and tentorium, endovascular neurointerventions and neurovascular surgery, as well as after subarachnoid hemorrhage [1-10]. However, until now, this phenomenon has only been described in adult patients during neurosurgical procedures.

We present, for the first time, a pediatric patient expressing a clear form of a TCR during revision of meningioma surgery.

## Case presentation

**Figure 1:**
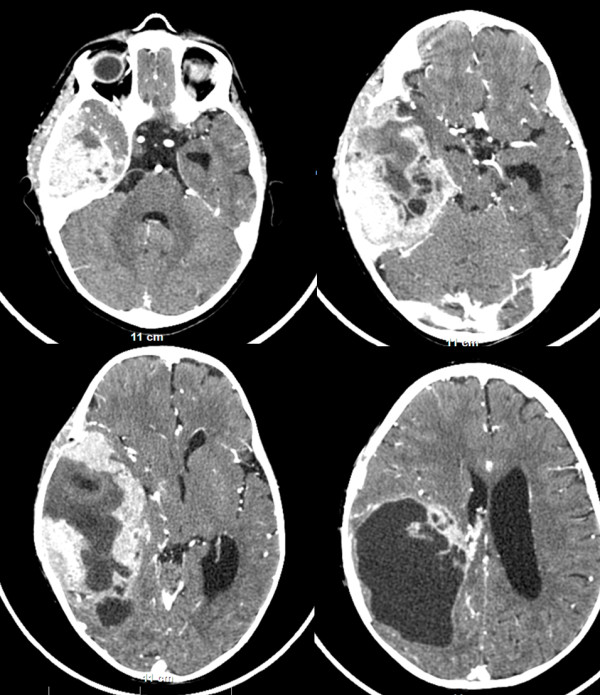
**A contrast-enhanced computed tomography scan two months after the first operation, revealing a large (110×61mm) right temporoparietal, contrast-enhancing mass, with a partial cystic component and significant brain compression.** The middle fossa and the parietal dura mater are invaded by the tumor. There is no significant brain edema.

An 18-month-old male Caucasian patient was admitted to our department with clinical presentation of partial motor seizures for left side extremities as well as signs and symptoms of intracranial hypertension (vomiting and lethargy). Two months before the current hospitalization, our patient underwent an operation in another institution with subtotal resection of a giant temporoparietal meningioma invading the middle fossa and parietal dura. The intervention at that time was terminated due to high blood loss. The histological result for the subtotally resected tumor was an anaplastic meningioma (World Health Organization grade III). He was receiving sodium valproate 30mg/kg. However, a computed tomography scan two months after the first operation revealed a large (110×61mm) right temporoparietal, contrast-enhancing mass, with a partial cystic component and significant brain compression (midline shift of 14mm) (Figure 1). His middle fossa and parietal dura mater were invaded by the tumor mass. There was no significant perifocal brain edema.

After discussion of the case and a review of the computed tomography and magnetic resonance imaging (MRI) scans, our patient was scheduled for elective revision surgery with the goal of total resection because of the prognostic relevance of the extent of surgical resection.

Our patient fasted for eight hours prior to surgery. Routine monitoring during surgery included electrocardiography, end-tidal concentration of CO_2_ and sevoflurane, and pulse oximetry. All hemodynamic parameters were monitored continuously and recorded throughout the neurosurgical procedure. Anesthesia was induced with sevoflurane (volume fraction 6% followed by fentanyl (1μg/kg) and atracurium (0.03mg/kg). After intubating his trachea, his lungs were mechanically ventilated (S/5 Aespire Config; Datex-Ohmeda Ins., Madison, WI, USA) with a mixture of air and O_2_ and sevoflurane (volume fraction 3%).

Our patient was placed supine on the operative table with his turned head placed on the three-point Mayfield head-holder rotated to the left side. After standard preparation of the operative field, a large horseshoe temporoparietal incision, repeating the one from the previous intervention, was made. After elevation of the bone flap, the revealed dura mater was found to be diffusely infiltrated by the tumor. After its excision the intracranial work was continued using the operative microscope. After intratumoral debulking, the tumor over the dura of the middle cranial fossa and tentorium was addressed. The tumor was dissected from the middle cranial fossa and the tentorium, during which time the anesthesiologist reported a significant sudden hypotension, from a mean arterial blood pressure (MABP) of 67mmHg to 30mmHg (55% drop), followed by a drop in heart rate from 140 beats/min to 110 beats/min (21% drop). These changes resolved upon cessation of the middle fossa dura manipulation. Ephedrine 0.01mg/kg was administered to counter the low MABP and the surgery continued. A second such episode occurred with manipulation of the middle fossa dura, with a drop in his MABP from 67mmHg to 43mmHg (36% drop), but with a tendency for tachycardia (170 beats/min) due to the previous application of ephedrine. Again, after a short cessation of the surgical intervention, his blood pressure parameters returned to baseline. Dopamine was administered on constant perfusion (3μg/kg/min) and a blood transfusion was initiated (8mL/kg).

Upon completion of the operation, our patient remained stable. No arrhythmias were documented postoperatively. His postoperative period was uneventful. The histological result showed again an anaplastic meningioma (World Health Organization grade III). Our patient was referred to a specialized pediatric oncology center.

Three months after the second intervention, a residual tumor mass (79×47mm) was discovered on control MRI and our patient underwent a further operation. No arrhythmias were documented during the intraoperative or postoperative period.

Two months after the third intervention, a ventriculoperitoneal catheter was introduced due to the development of hydrocephalus. Again no arrhythmias were documented during the intraoperative and postoperative period.

Eight months after the first intervention a follow-up MRI study was performed. A large recurrent tumor mass (64×21mm) was discovered invading his middle cranial fossa, tentorial edge and Meckel’s cave, with posterior fossa extension. A large postoperative cyst (34×45mm) was also revealed. Because our patient’s condition was stable, the decision was made not to surgically attack the tumor and to continue close follow-up radiological studies. Our patient`s follow-up regarding the TCR remained uneventful.

## Discussion

To the best of our knowledge, this is the first report of a central TCR in a pediatric patient during an open neurosurgical procedure. The current case includes all definitions of the TCR as summarized previously by the senior author [1,4,5]. Studying the TCR in the pediatric population is important. It has been suggested that the TCR may behave differently in pediatric patients compared with adult patients because of the persistence of the diving reflex into the first months of life [5].

A careful literature review revealed three other reported cases of TCR initiation in children [11-13], however they referred to the peripheral form of TCR. Stavrinou *et al*. [13] report the case of a 10-year-old girl, selected for a stereotactic biopsy of her fourth ventricle and pontine mass, who experienced a sudden drop in her blood pressure and heart rate after placement of a stereotactic frame, which resolved after removal of the frame. The provoking factor for the TCR was attributed to incorrect pin placement over the supraorbital nerve, a branch of the ophthalmic nerve. Puri *et al*. [12] and Potti *et al*. [11] describe the occurrence of the TCR during embolization of nasopharyngeal angiofibromas (see Table 1); these are again examples of the peripheral form of TCR.

**Table 1:** Description of central and peripheral pediatric trigeminocardiac reflex case reports in the literature

**Author**	**TCR type**	**Case description**	**TCR occurrence and management**
Potti *et al*. [11]	Peripheral	Percutaneous embolization with DMSO of a juvenile nasopharyngeal angiofibroma.	Bradycardia and asystole (30 second duration), resolved after cessation of the procedure and administering anticholinergic drugs.
Puri *et al*. [11]	Peripheral	Percutaneous embolization with DMSO of a juvenile nasopharyngeal angiofibroma in a 10-year-old boy.	Bradycardia resolved after cessation of the procedure and administering anticholinergic drugs.
Stavrinou *et al*. [13]	Peripheral	Stereotactic biopsy of fourth ventricle and pontine tumor in a 10-year-old Caucasian girl.	Incorrect pin holder placement over the supraorbital nerve, managed by cessation of the intervention.
Spiriev *et al*.	Central	Middle fossa meningioma on an 18-month-old Caucasian boy.	Surgical manipulation of the middle fossa dura and tentorium. TCR managed by cessation of the stimulus and ephedrine administration due to very low mean arterial blood pressure.

DMSO: dimethylsulfoxide; TCR: trigeminocardiac reflex.

A higher incidence of cardiovascular disturbances in children is well known to occur during ophthalmic surgery [8,9]. The incidence of the underlying OCR has been variously reported as 14% to 90%, depending on the premedication, anesthetic agent, and the definition of OCR [8,9]. There are seven reported rare cases of mortality resulting from initiation of the OCR in pediatric patients during strabismus surgery [8,9]. These and other reports clearly underline the importance of this first description of a central TCR in the pediatric population, because – from the data above – it can be suggested that the behavior of the TCR in pediatric patients might be more aggressive than in adult patients. To prove this hypothesis, larger case series are necessary. Nevertheless, it is now well known that the intraoperative occurrence of the central form of TCR significantly influences the outcome in selected group of patients [6,14], as is shown in surgery for vestibular schwannoma. Taking into account all these data, TCR represents a substantial risk factor during routine neurosurgical procedures. Future research should focus on possible predisposing and provoking factors and prevention.

The anatomical substrate for the TCR arises in the sensory afferent fibers in the trigeminal nerve, including those in the ethmoidal nerve, which project via the Gasserian ganglion to the sensory nucleus of the trigeminal nerve [1,4,5]. The pathway continues from the ventral trigeminal nucleus through the short internuncial nerve fibers in the reticular formation in the brain stem to finally synapse on efferent premotor parasympathetic cardioinhibitory neurons in the nucleus ambiguus [1,4,5]. In our case report, the TCR was initiated by surgical manipulations around the dura in the middle cranial fossa and tentorium. According to the studies of Penfield and McNaughton [15] the nervus tentorii, a recurrent branch of the ophthalmic branch of the trigeminal nerve, bilaterally innervates the tentorium cerebelli. The meningeus medius, a branch of V2, is described to run with the anterior branch of the middle meningeal artery to innervate the middle fossa dura [15], which in our case was considered to elicit the TCR. However laboratory investigations, clinical experience with the TCR and knowledge of the anatomical distribution of the trigeminal nerve support the assumption that stimulation of any sensory branch of the fifth cranial nerve may lead to severe bradycardia and/or arterial hypotension [1-10].

It has already been shown by us and others that intraoperative direct nerve stimulus is the most important provoking factor for TCR: during surgery, abrupt and sustained traction is more reflexogenic than smooth and gentle traction [1,4,5,14]. However there is an important factor which needs to be mentioned in our particular case - that it was a revision surgery. Postoperative changes, such as scaring and aseptic inflammation, could lead to sensitization of the trigeminal afferents in the dura mater, as previously demonstrated by our group [10]. Meningeal afferents are thought to become activated only under potentially harmful or pathological conditions. However, although the dural afferent population does not appear to mediate distinct sensory modalities, it shows a pattern of variation in mechanosensitivity as a function of conduction velocities. Such exaggerated mechanical sensitivity and manipulation of the dura mater could have played a role in the initiation of TCR in our case.

## Conclusions

We present here, a unique case of central TCR in a pediatric patient that occurred during a revision operation of a giant meningioma. It shows that the TCR occurs in all age groups; even so, we do not know much about the incidence and the behavior of the TCR in the pediatric age group. From data regarding the OCR, a peripheral subform of the TCR, it seems that the TCR is more prone and more aggressive in pediatric than in adult patients. But these hypotheses have to be proven by further research.

## Consent

Written informed consent was obtained from the patient’s legal guardian for publication of this case report and accompanying images. A copy of the written consent is available for review by the Editor-in-Chief of this journal.

## Competing interests

The authors declare that they have no competing interests.

## Authors’ contributions

TS and BS wrote the article. TS and LL collected the data. BS interpreted and analyzed the data. SK and CT performed the operation and the patient’s treatment and provided substantial information regarding the patient’s case and were therefore major contributors to writing the manuscript. NS and CK provided some specific and general ideas that initiated the work and helped to finish the work. Without both contributions, this report would not have been possible. NS made substantial corrections to the manuscript. All authors read and approved the final manuscript.
